# Supplementary material for: QTL Analysis of β-Glucan Content and Other Grain Traits in a Recombinant Population of Spring Barley
Source: Int J Mol Sci. 2024 Jun 7;25(12):6296. doi: 10.3390/ijms25126296 (PMC11204098; doi:10.3390/ijms25126296)
Supplement: Supplementary file 1 [file ijms-25-06296-s001.zip › ijms-3026939-supplementary/Supplementary - Correlation Analysis.html]

Results: Program 1


# Results: Program 1

# The Corr Procedure

# Variables Information

|  |  |
| --- | --- |
| 8 Variables: | Betaglucan Protein Starch Lipid Ash Calcium Phosphorus Sodium |

# Pearson Correlations

| Pearson Correlation Coefficients, N = 183 Prob > |r| under H0: Rho=0 | | | | | | | | |
| --- | --- | --- | --- | --- | --- | --- | --- | --- |
|  | Betaglucan | Protein | Starch | Lipid | Ash | Calcium | Phosphorus | Sodium |
| Betaglucan  Betaglucan | 1.00000 | -0.14525  0.0498 | -0.29372  <.0001 | 0.69341  <.0001 | -0.15224  0.0397 | 0.25811  0.0004 | 0.20106  0.0063 | 0.14546  0.0494 |
| Protein  Protein | -0.14525  0.0498 | 1.00000 | -0.23916  0.0011 | -0.04773  0.5211 | 0.65317  <.0001 | 0.13132  0.0764 | 0.00582  0.9376 | 0.03847  0.6052 |
| Starch  Starch | -0.29372  <.0001 | -0.23916  0.0011 | 1.00000 | 0.23749  0.0012 | -0.66636  <.0001 | 0.57925  <.0001 | 0.69582  <.0001 | 0.64614  <.0001 |
| Lipid  Lipid | 0.69341  <.0001 | -0.04773  0.5211 | 0.23749  0.0012 | 1.00000 | -0.37930  <.0001 | 0.70560  <.0001 | 0.65794  <.0001 | 0.53479  <.0001 |
| Ash  Ash | -0.15224  0.0397 | 0.65317  <.0001 | -0.66636  <.0001 | -0.37930  <.0001 | 1.00000 | -0.45215  <.0001 | -0.56545  <.0001 | -0.54006  <.0001 |
| Calcium  Calcium | 0.25811  0.0004 | 0.13132  0.0764 | 0.57925  <.0001 | 0.70560  <.0001 | -0.45215  <.0001 | 1.00000 | 0.97767  <.0001 | 0.84618  <.0001 |
| Phosphorus  Phosphorus | 0.20106  0.0063 | 0.00582  0.9376 | 0.69582  <.0001 | 0.65794  <.0001 | -0.56545  <.0001 | 0.97767  <.0001 | 1.00000 | 0.87450  <.0001 |
| Sodium  Sodium | 0.14546  0.0494 | 0.03847  0.6052 | 0.64614  <.0001 | 0.53479  <.0001 | -0.54006  <.0001 | 0.84618  <.0001 | 0.87450  <.0001 | 1.00000 |

# Scatter Plot Matrix
