# Supplementary material for: QTL Analysis of β-Glucan Content and Other Grain Traits in a Recombinant Population of Spring Barley
Source: Int J Mol Sci. 2024 Jun 7;25(12):6296. doi: 10.3390/ijms25126296 (PMC11204098; doi:10.3390/ijms25126296)
Supplement: Supplementary file 1 [file ijms-25-06296-s001.zip › ijms-3026939-supplementary/Supplementary Figure S1.pdf]

Supplementary Figure S1

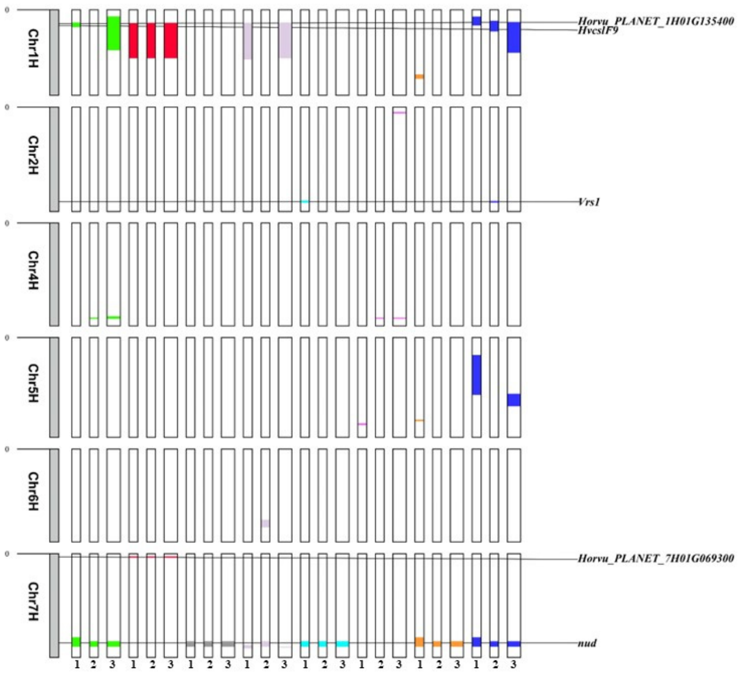

Figure S1. Graphical representation of QTLs across chromosomes. Known genes co-localized with our QTLs, and the putative candidate genes, have been reported. QTLs obtained using all environments tested (2021, named as '1'; 2022 as '2') and in the multi environment (MEnv, '3') are reported. The colors represent the different phenotypic traits: green stands for ash, red for β-glucan, grey for calcium, light purple for lipid, light blue for phosphorus, pink for protein, orange for sodium and blue for starch.
